# Early Postoperative Outcomes of Breast Cancer Surgery in a Developing Country

**DOI:** 10.7759/cureus.9941

**Published:** 2020-08-22

**Authors:** Farrukh H Rizvi, Muhammad Kashif Khan, Talal Almas, Muneeb Ullah, Adil Shafi, Muhammad Faisal Murad, Aabid Ali, Faisal Nadeem

**Affiliations:** 1 Surgery, Mukhtar A. Sheikh Hospital, Multan, PAK; 2 Surgical Oncology, Federal Government Poly Clinic (Post Graduate Medical Institute), Islamabad, PAK; 3 Surgical Oncology, Maroof International Hospital, Islamabad, PAK; 4 Internal Medicine, Royal College of Surgeons in Ireland, Dublin, IRL; 5 General Surgery, Maroof International Hospital, Islamabad , PAK; 6 General Surgery, Maroof International Hospital, Islamabad, PAK; 7 Laparoscopic Surgery, Maroof International Hospital, Islamabad, PAK

**Keywords:** breast cancer outcomes, modified radical mastectomy (mrm), breast conservation therapy

## Abstract

Background

Breast cancer remains the most common cause of cancer related mortality amongst women in Pakistan. Postoperative complications can demoralize the patients and potentially delay adjuvant treatment, leading to adverse outcomes. The overarching aim of the study is to delineate the early postoperative outcomes of breast cancer surgery in Pakistan.

Materials and Methods

A retrospective study involving patients who underwent breast cancer surgery from June 2016 to December 2019 was conducted. Perioperative morbidities (30 days) were evaluated and documented. The results obtained were analyzed using the SPSS 23 software (IBM Corporation, Armonk, NY).

Results

A total of 94 patients were included in the study, with the mean age of 50±12.8 years. Breast conserving surgery was performed in 32% (n=31) of the patients, while the remaining 68% (n=63) underwent modified radical mastectomy. The most common complications were seroma formation, flap necrosis and hematoma formation and were observed in 5.3% (n=5), 4.3% (n=4) and 3.2% (n=3) of the patients, respectively.

Conclusion

Early postoperative complications can delay the commencement of adjuvant systemic therapy required for further management of breast cancer. These complications elicit equally grave consequences for patients undergoing breast conserving surgery and modified radical mastectomy.

## Introduction

Breast cancer is the second most common malignancy worldwide and boasts the highest incidence rate among all types of cancers [1,2]. A delay in seeking care is associated with poor survival outcomes [2]. Notably, there is a significant variation in the five-year survival rates worldwide; in the developed world, for instance, the five-year survival rate approaches 83.2% while that in developing countries such as Brazil and India it hovers at 58% and 52.1%, respectively [3,4]. In Pakistan, patients present late either due to a lack of awareness about the disease or due to access merely to rudimentary resources. Additional reasons, such as fear of an impending surgery and chemotherapy, also explain the dilatory presentation. Consequently, breast conservation is not pragmatic in a myriad of breast cancer patients.

Surgical treatment depends on a plethora of factors, including the stage at the time of presentation, the availability of resources and patient preference [5-8]. Furthermore, the diagnosis and the treatment for breast cancer have significant psychosocial implications, which can be further compounded if postoperative complications ensue. Postoperative complications can be observed early and include wound infection, seroma formation, hematoma and flap necrosis [4,9]. Late complications include shoulder stiffness, brachial plexopathy and psychosocial disturbances [6-8]. Unfortunately, many risk factors for the development of postoperative complications are not modifiable in the time scale between the initial diagnosis and the eventual surgery.

The objective of the present study is to elucidate our experience with various surgical choices in operable breast cancer patients and the short-term outcomes that subsequently develop in our patient cohort. There is a scarcity of data from Pakistan delineating the outcomes of breast conservation surgery (BCS) since this option is not frequently employed due to the lack of resources and suitable patient population.

## Materials and methods

We retrospectively reviewed patients who underwent breast cancer surgery between June 2016 and December 2019 at Maroof International Hospital, Islamabad, Pakistan. All patients underwent triple assessment for their breast lumps along with clinical staging of the disease using ultrasound liver, chest radiograph and bone scan. Patients with stage IV disease and those who required reconstruction following modified radical mastectomy (MRM) were excluded from the study. Patients were staged according to American Joint Committee on Cancer (AJCC) breast cancer staging system 8 and divided into early breast cancer (EBC), comprising of stages IA, IB, IIA and IIB, and locally advanced breast cancer (LABC), consisting of stages IIIA and IIIB. All the patients were discussed in a multidisciplinary team (MDT) meeting, involving the surgical oncologist, clinical oncologist, radiologist and histopathologist. Thereafter, the most apt treatment plan was devised and offered to the patients. Patients with EBC were assessed for feasibility of BCS. Patients were educated about the disease and treatment options before informed consent was obtained. Factors affecting wound healing, including age, hypertension, diabetes, ischemic heart disease, antiplatelet therapy and neoadjuvant systemic therapy, were noted. Due to an absence of the facility to conduct sentinel lymph node biopsy, a level II axillary lymph node dissection was performed in all of the patients. During surgery, flap dissection was performed using diathermy and hemostasis was secured using diathermy and sutures. Two closed suction drains were placed, one in the axilla and the other under the flap in MRM. A single drain was placed in axilla in cases of BCS. Flap drain was subsequently removed at the time of discharge, while the axillary drain was removed on future outpatient visits given that the discharge had plummeted to 30 ml in the last 24 hours. 

Postoperative morbidity was recorded as any abnormal event in first 30 days after the surgery. Patients were reviewed in clinic on the postoperative days 5 and 14, and then at six weeks after surgery. The parameters of early outcomes, such as wound infection, seroma formation, skin flap necrosis, hematoma, re-exploration, blood transfusion and length of hospital stay, were noted. All the patients were referred to an oncologist for adjuvant treatment. Patients received chemotherapy depending upon the MDT decision either before or after the surgery. Those who underwent BCS were referred to a radiation oncologist for radiation to the breast. Patients with hormone receptor positive disease were then started on endocrine treatment depending on their menopausal status. Clearance from the institutional ethical committee was duly obtained for this study. The data collected were eventually analyzed using the SPSS 23 software (IBM Corporation, Armonk, NY).

## Results

**Figure 1:**
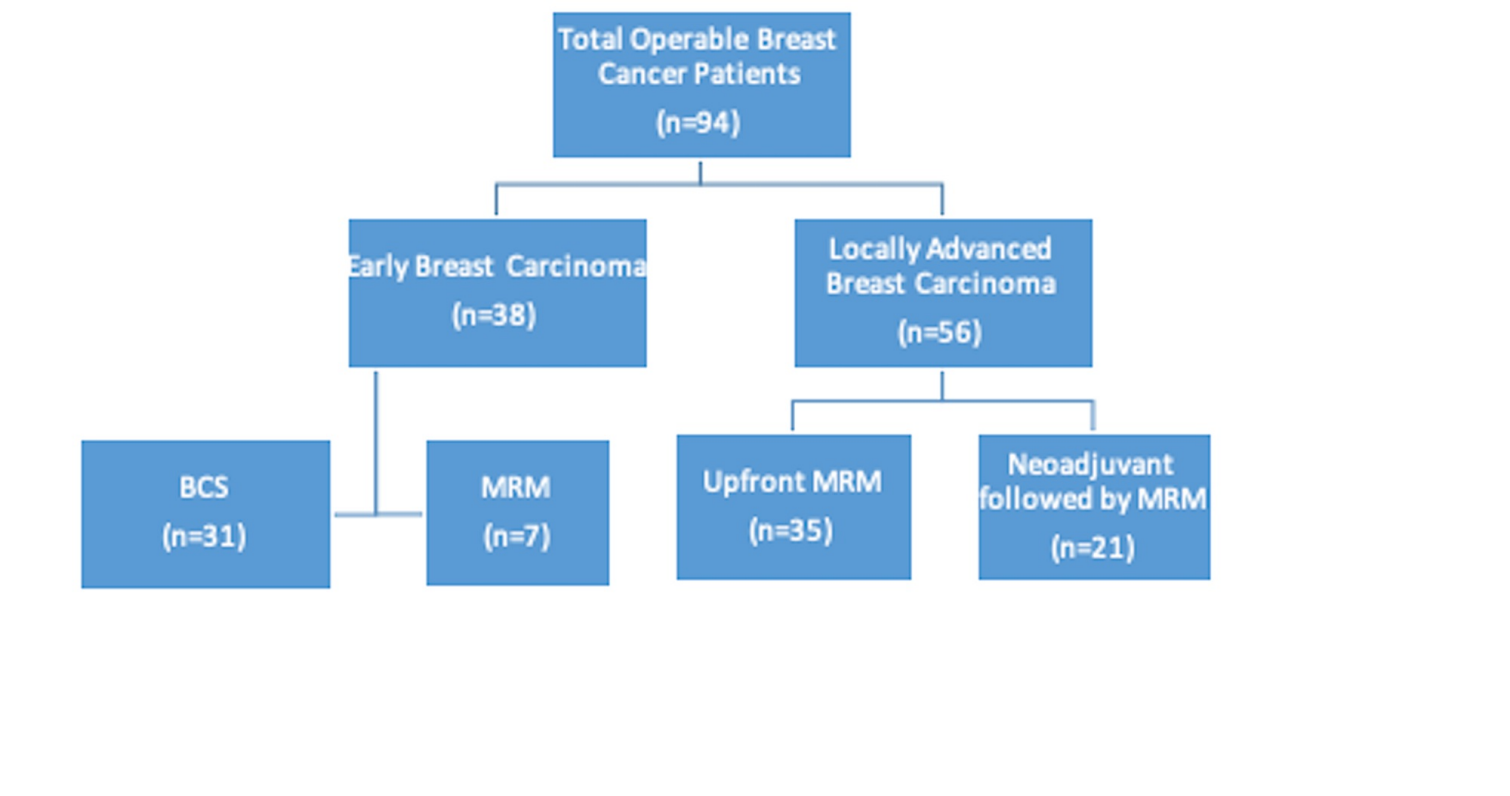
The treatment modalities employed in breast cancer patients. BCS, breast conservation surgery; MRM, modified radical mastectomy.

Out of a total of 94 patients, 40.4% (n=38) had EBC, while 59.6% (n=56) had LABC. The various treatment modalities opted in these patients are delineated in Figure 1. 

The pathological distribution with pertinence to the surgical modality opted is further shown in Table 1. 

**Table 1:** The particular tumor pathology for both the surgical modalities employed (p value = 0.729).

Histopathology			
Procedure	Invasive carcinoma not otherwise specified (NOS)	Invasive lobular carcinoma	Total
Breast conserving surgery	30	1	31
Modified radical mastectomy	60	3	63
Total	90	4	94

Of the patients included, 67% (n=63) underwent MRM, while the rest underwent BCS. The comorbidities of the patients with reference to the surgical modality employed are further highlighted in Table 2. 

**Table 2:** Pre-treatment characteristics of the patients studied.

Characteristics	Modified radical mastectomy (N=63)	Breast conservation surgery (N=31)	Total surgeries (N=94)	P-value
Diabetes mellitus	16	3	19	0.74
Hypertension	20	8	28	0.54
Ischemic heart disease	6	3	9	0.98
Asthma	2	2	4	0.98
Thyroid disease	0	3	3	0.12
Antiplatelet therapy	7	4	11	0.79

Lobular carcinoma was noted in merely 5.3% (n=5) of the patients, while the rest of the tumors were invasive carcinomas not otherwise specified (NOS). All patients undergoing BCS were those with EBC (p-value = 0.001) (Table 3).

**Table 3:** A comparison of the clinical stages in patients undergoing breast conservation surgery and modified radical mastectomy.

Procedure	Stage IA	Stage IIA	Stage IIB	Stage IIIA	Stage IIIB	Total
Breast conservation surgery	5	18	8	0	0	31
Modified radical mastectomy	0	6	2	43	12	63
Total	5	24	10	43	12	94

Pertinently, the mean age of the patients was 51.79±12.65 years and the mean duration of surgery was 1.7±0.47 hours. The mean hospital stay was 2.1±0.88 days. The mean number of axillary nodes retrieved was 16.82±6.8 nodes, while the mean number of positive nodes was 2.63±3.5 nodes. The frequencies of early complications for both BCS and MRM are delineated in Table 4. 

**Table 4:** The postoperative outcomes of breast cancer surgery. BCS, breast conservation surgery; MRM, modified radical mastectomy.

Outcomes		MRM (N=63)	BCS (N=31)	Total (N=94)	P-value
Seroma		4	1	5	0.52
Flap necrosis	Partial thickness	3	1	4	0.72
	Full thickness	0	0	0	
Wound infection		1	0	1	0
Hematoma		3	0	3	0.48
Re-exploration		2	0	2	0.21
Re-admission		2	0	2	0.31

As can be noted, there is no significant difference in early complications when comparing the BCS and MRM groups. Furthermore, the outcomes of upfront surgery were compared with those of neoadjuvant chemotherapy and are depicted in Table 5. 

**Table 5:** Early outcomes in patients undergoing neoadjuvant chemotherapy and upfront surgery.

Outcomes		Upfront surgery (N=69)	Neoadjuvant chemotherapy (N=25)	Total (N=94)	P-value
Seroma		3	2	5	0.48
Flap necrosis	Partial thickness	3	1	4	0.94
	Full thickness	0	0	0	
Wound infection		1	0	1	0.95
Hematoma		2	1	3	0.78
Re-exploration		2	0	2	0.39
Re-admission		2	0	2	0.39

## Discussion

Surgery for breast cancer remains an exceedingly pivotal undertaking in a woman’s life. Breast cancer treatment has evolved in the last few decades, with more and more patients now undergoing breast conservation with combined modality treatment [10-12]. Most of these patients adhere to a therapeutic regimen that encompasses a concoction of chemotherapy and radiotherapy [13]. While stringent adherence to such regimens can portend favorable surgical outcomes, a delay in treatment due to any comorbidity can adversely impact the patients’ overall survival [14]. In Pakistan, a vast majority of the females are reluctant to seeking medical advice for breast-related issues owing primarily to the insular and parochial social norms present. There is thus an unmet need in Pakistan for breast cancer patients to be managed psychosocially, ranging from therapy and counseling sessions to social support and referral to proper healthcare facility. While surgeons vie to perform surgeries that demonstrate good postoperative outcomes, an interplay of factors, including patient education, is often necessary for yielding optimal cancer-related outcomes [6,12,15]. 

Various factors have been associated with the quality of postoperative outcomes after breast surgery. These factors include tumor factors (tumor size, lymph node status), patient factors (age, weight, diabetes mellitus, hypertension, smoking) and surgical factors (use of electrocautery for flap dissection, length of operation time) [5,9,14]. Wound-related complications can cause significant delays in the commencement of adjuvant therapy, often resulting in aesthetic compromise, patient distress and financial loss [12]. The National Institute for Health and Care Excellence (NICE) espouses the notion that it is pivotal to initiate adjuvant therapy within 31 days of completion of surgery [16]. Additionally, the European Society of Medical Oncology guidelines further indicate that treatment should ideally start within two to six weeks of surgery [12,15,16]. Nevertheless, oncologists remain reluctant to administering chemoradiotherapy in patients with compromised recovery or delayed wound healing. Further complicating this reluctance is the notion that in our part of the world, most tumors are relatively advanced at presentation [12]. A delay in adjuvant treatment can potentially elicit grave implications for overall and disease-free survival. A plethora of studies elucidate the detrimental impact that delayed adjuvant chemotherapy can yield [14-18]. In our study, upfront surgery was performed in 69 candidates; out of which only 2 patients received delayed adjuvant treatment beyond six weeks. Patients undergoing BCS had similar outcomes after surgery as compared to those undergoing MRM; however, merely a smaller number of patients underwent BCS owing to their advanced disease stage at presentation and a reluctance on the part of the patients to undergo radiation treatment. Neoadjuvant therapy allows direct and early observation of the response to treatment; however, these patients should be continuously followed to monitor for potential disease progression [16-19]. There is an increasing trend of offering neoadjuvant systemic therapy in patients with LABC based on the 30%-39% pathological complete response (pCR) in patients with aggressive histology [16]. Pertinently, we did not observe an increased incidence of complications in patients who received neoadjuvant chemotherapy, a notion that largely corroborates the findings elucidated by the international data [17].

The most frequent complication observed in our patients was seroma formation. In the reported literature, the incidence of seroma demonstrates a wide variation due to differences in definitions practices involving drain placement [17,18]. While a miniscule degree of fluid collection occurs in most of the patients, it is only appreciated in instances where there is a significant amount of fluid. Furthermore, the fluid aggregation becomes severe or symptomatic enough to be aspirated in only a minority of the patients [19-21]. In our practice, an axillary drain was placed in all patients and the patients were subsequently discharged with the drain left in place. The drain was then removed on a future OPD visit. In our study, aspiration of the surgical wound was warranted in only five patients. While this complication routinely ensues after breast cancer surgery, it is easily managed and does not usually result in delays in the commencement of adjuvant treatment. 

Skin flap necrosis is a significant but avoidable problem that can ensue in the aftermath of breast cancer surgery [12]. A detailed history regarding previous ischemic heart disease, cerebrovascular accident and diabetes must be obtained, and the patient must be assessed clinically prior to surgery [18]. With pertinence to the operating techniques employed, the skin flap thickness, tightness of the closure and the rational use of diathermy remain the focal factors. After the assessment of subcutaneous body fat, the oncological plane between the subcutaneous fat and the breast parenchyma is exploited to preserve blood supply of the flaps [12-14]. Generally, the skin flap thickness should be around 6-8 mm [12]. We use Mayo clinic skin ischemia and necrosis score to classify it into partial or full thickness necrosis [19]. We did not encounter full thickness necrosis in any of our patients, while partial thickness necrosis was noted in three patients who underwent MRM and in one patient who underwent BCS. The BCS patient who had partial skin necrosis was actually a patient who had level II oncoplastic surgery resulting in an inverted T-shaped scar. All these patients were managed on an OPD basis with dressings. Furthermore, the incidence of surgical site infection after breast surgery varies across studies between 0.1% and 12.5% [18]. Wound infection rate in our study was 1.06% and was thus significantly lower when compared to other studies conducted in Pakistan, which have divulged infection rates in the range of 5.4% to 11.4% [7,8,11]. Surgical site infections were managed conservatively using oral antibiotics and local antiseptic dressings.

In the present study, the incidence of hematoma formation was 3.19% (n=3), which is in accordance with the international data. One of these patients was managed on an OPD basis, while the remaining two patients required re-admission and re-exploration. This is a significant complication since, in both of these patients, the adjuvant treatment was delayed beyond six weeks. Meticulous surgery in the relatively bloodless oncological plane, and hemostasis at the end of the surgery can help circumvent his complication. None of the patients required blood transfusion during the surgery; however, three patients were transfused blood after the surgery. A detailed assessment and management of comorbidities, meticulous surgical technique and good postoperative care are the main factors through which we can ensure smooth recovery after surgery for breast cancer. 

## Conclusions

Our data from an evolving cancer surgery setup in a developing country suggest that a higher percentage of our patients ended up undergoing mastectomies while our morbidity rates for both BCS and MRM are low and comparable, with no statistically significant differences. 
